# Risk of Advanced Neoplasia in First-Degree Relatives with Colorectal Cancer: A Large Multicenter Cross-Sectional Study

**DOI:** 10.1371/journal.pmed.1002008

**Published:** 2016-05-03

**Authors:** Enrique Quintero, Marta Carrillo, Maria-Liz Leoz, Joaquin Cubiella, Carla Gargallo, Angel Lanas, Luis Bujanda, Antonio Z. Gimeno-García, Manuel Hernández-Guerra, David Nicolás-Pérez, Inmaculada Alonso-Abreu, Juan Diego Morillas, Francesc Balaguer, Alfonso Muriel, on behalf of the Oncology Group of the Asociación Española de Gastroenterología (AEG)

**Affiliations:** 1 Servicio de Gastroenterología, Hospital Universitario de Canarias, Instituto Universitario de Tecnologías Biomédicas (ITB) & Centro de Investigación Biomédica de Canarias (CIBICAN), Departamento de Medicina Interna, Universidad de La Laguna, San Cristóbal de La Laguna, Tenerife, España; 2 Servicio de Gastroenterología, Hospital Clínic de Barcelona, Centro de Investigación Biomédica en Red de Enfermedades Hepáticas y Digestivas (CIBERehd), Institut d’Investigacions Biomediques August Pi i Sunyer (IDIBAPS), Universidad de Barcelona, Barcelona, Cataluña, España; 3 Servicio de Gastroenterología, Complejo Hospitalario Universitario de Ourense, Ourense, Galicia, España; 4 Servicio de Gastroenterología, Hospital Clínico Universitario de Zaragoza, Zaragoza, España; 5 Servicio de Gastroenterología, Hospital Donostia-Instituto Biodonostia, Centro de Investigación Biomédica en Red de Enfermedades Hepáticas y Digestivas (CIBERehd), Universidad del País Vasco UPV-EHU, San Sebastián, España; 6 Departmento de Gastroenterología, Hospital Clínico Universitario San Carlos, Madrid, España; 7 Hospital Universitario Ramón y Cajal, Unidad de Bioestadística C, IRYCIS, Madrid, Centro de Investigación Biomédica en Red de Epidemiología y Salud Pública (CIBERESP), Madrid, España; McGill University, CANADA

## Abstract

**Background:**

First-degree relatives (FDR) of patients with colorectal cancer have a higher risk of developing colorectal cancer than the general population. For this reason, screening guidelines recommend colonoscopy every 5 or 10 y, starting at the age of 40, depending on whether colorectal cancer in the index-case is diagnosed at <60 or ≥60 y, respectively. However, studies on the risk of neoplastic lesions are inconclusive. The aim of this study was to determine the risk of advanced neoplasia (three or more non-advanced adenomas, advanced adenoma, or invasive cancer) in FDR of patients with colorectal cancer compared to average-risk individuals (i.e., asymptomatic adults 50 to 69 y of age with no family history of colorectal cancer).

**Methods and Findings:**

This cross-sectional analysis includes data from 8,498 individuals undergoing their first lifetime screening colonoscopy between 2006 and 2012 at six Spanish tertiary hospitals. Of these individuals, 3,015 were defined as asymptomatic FDR of patients with colorectal cancer (“familial-risk group”) and 3,038 as asymptomatic with average-risk for colorectal cancer (“average-risk group”). The familial-risk group was stratified as one FDR, with one family member diagnosed with colorectal cancer at ≥60 y (*n* = 1,884) or at <60 y (*n* = 831), and as two FDR, with two family members diagnosed with colorectal cancer at any age (*n* = 300). Multiple logistic regression analysis was used for between-group comparisons after adjusting for potential confounders (age, gender, and center). Compared with the average-risk group, advanced neoplasia was significantly more prevalent in individuals having two FDR with colorectal cancer (odds ratio [OR] 1.90; 95% confidence interval [CI] 1.36–2.66, *p* < 0.001), but not in those having one FDR with colorectal cancer diagnosed at ≥60 y (OR 1.03; 95% CI 0.83–1.27, *p* = 0.77) and <60 y (OR 1.19; 95% CI 0.90–1.58, *p* = 0.20). After the age of 50 y, men developed advanced neoplasia over two times more frequently than women and advanced neoplasia appeared at least ten y earlier. Fewer colonoscopies by 2-fold were required to detect one advanced neoplasia in men than in women.

Major limitations of this study were first that although average-risk individuals were consecutively included in a randomized control trial, this was not the case for all individuals in the familial-risk cohort; and second, the difference in age between the average-risk and familial-risk cohorts.

**Conclusions:**

Individuals having two FDR with colorectal cancer showed an increased risk of advanced neoplasia compared to those with average-risk for colorectal cancer. Men had over 2-fold higher risk of advanced neoplasia than women, independent of family history. These data suggest that screening colonoscopy guidelines should be revised in the familial-risk population.

## Abstract 2

Quintero and colleagues show that relatives of colorectal cancer patients have a higher risk of themselves developing the disease, as well as neoplasias. Screening guidelines should be accordingly adjusted.

## Editors' Summary

**Background:**

Colorectal cancer kills about 700,000 people worldwide every year. This common cancer develops when one of the epithelial cells that lines the colon (the final part of the digestive system; also known as the large intestine or large bowel) or the rectum (the lower end of the colon) acquires genetic changes that allow it to divide uncontrollably to form a tumor (lump), to invade surrounding tissues, and to move around the body (metastasize). Symptoms of colorectal cancer include weight loss, blood in the stool, and changed bowel habits. Treatments for the condition include surgery, chemotherapy, and radiation. As with all types of cancer, these treatments are more likely to be successful if started when the tumor is small and before it has invaded surrounding tissues. Consequently, many countries run screening programs to detect the earliest signs of colorectal cancer in apparently healthy people.

**Why Was This Study Done?:**

One of the methods used to screen for colorectal cancer is colonoscopy—visual examination of the colonic lining using a tube-like instrument that is inserted through the rectum into the colon. Colonoscopy also detects adenomas (polyps), benign tumors that lack the ability to invade or metastasize. It is important to find and remove adenomas because these growths can develop into colorectal cancers. In the United States, colonoscopy is recommended every 10 y for everyone in the general population over 50 y old. However, the first-degree relatives (parents, siblings, or child) of patients with colorectal cancer have a higher risk of developing colorectal cancer than people in the general population. So, current screening guidelines in the US and elsewhere recommend colonoscopy every 5 or 10 y from age 40 for people who have a first-degree relative diagnosed with colorectal cancer, depending on whether the relative’s colorectal cancer was diagnosed before or after the age of 60, respectively. Notably, no large-scale studies have compared the risk of adenomas in symptom-free people with first-degree relatives with colorectal cancer with the risk in symptom-free average-risk people. This large multicenter cross-sectional study (a study that looks at a single time point) aims to remedy this deficit in the evidence base.

**What Did the Researchers Do and Find?:**

The researchers enrolled 3,015 first-degree relatives of patients with colorectal cancer and 3,038 average-risk individuals, all of whom were undergoing their first screening colonoscopy in Spain. They divided the familial-risk cohort into two groups. The 1-FDR group comprised people with one first-degree relative diagnosed with colorectal cancer at ≥60 y or at <60 y. The 2-FDR group comprised individuals with two first-degree relatives diagnosed with colorectal cancer at any age. They then compared the risk of advanced neoplasia (three or more non-advanced adenomas, advanced adenoma, or invasive cancer) in the different groups after adjusting for age and sex (confounding factors that affect an individual’s risk of colorectal cancer). Advanced neoplasia was more prevalent (present in a higher proportion of the population) in the 2-FDR group than in average-risk individuals; individuals in the 2-FDR group were nearly twice as likely to have advanced neoplasia as average-risk individuals. Compared to average-risk individuals, individuals with 1-FDR diagnosed at ≥60 y and at <60 y had no increased risk of advanced neoplasias. Notably, after age 50, men developed advanced neoplasias over 2-fold more frequently than women, and advanced neoplasias appeared at least 10 y earlier in men than in women.

**What Do These Findings Mean?:**

These findings suggest that the risk of advanced neoplasia is markedly increased in individuals having two first-degree relatives with colorectal cancer compared to average-risk individuals but not in individuals having only one affected first-degree relative. They also suggest that men have a 2-fold higher risk of advanced neoplasia than women, independent of family history. Several aspects of the study design may affect the accuracy of these findings. For example, the average-risk study participants were older than the participants with first-degree relatives with colorectal cancer, and different approaches were used to enroll the two sets of participants, which may have introduced some bias. Nevertheless, these findings suggest that the guidelines for screening for colorectal cancer should be revised. Specifically, they suggest that men with two first-degree relatives with colorectal cancer may benefit from early screening colonoscopy but that the screening strategy recommended for the average-risk population may be appropriate for individuals with only one first-degree relative with colorectal cancer.

**Additional Information:**

This list of resources contains links that can be accessed when viewing the PDF on a device or via the online version of the article at http://dx.doi.org/10.1371/journal.pmed.1002008.

- The US National Cancer Institute provides information for patients about all aspects of colorectal cancer, including information on tests to detect colorectal cancer; it also provides more detailed information about the condition and about screening for health professionals (in English and Spanish)
- The United Kingdom National Health Service Choices website has information about colorectal cancer (including some personal stories) and information on screening for colorectal cancer
- The not-for-profit organization Cancer Research UK also provides information about colorectal cancer
- Details of colorectal cancer screening guidelines in the US and in the UK are available
- MedlinePlus provides links to further resources about colorectal cancer (in English and Spanish) and has a page about colorectal adenomas

## Introduction

Clinical guidelines recommend more intensive surveillance in first-degree relatives (FDR) of patients with colorectal cancer than in average-risk individuals, as their risk of developing colorectal cancer is 2- to 4-fold higher [1–4]. Screening in this population is recommended from the age of 40 or 10 y before the youngest case in the immediate family, since the disease tends to develop about 10 y earlier in FDR than in the general population [5]. Colonoscopy every 5 [3,6–8] or 10 [3] y, depending on the number of relatives affected and age at cancer diagnosis, is the predominant screening strategy for these individuals, as it is the most effective procedure to detect and remove advanced adenomas. However, this recommendation is not supported by prospective studies.

Low-penetrance genetic alterations may favor earlier development of advanced adenomas or accelerate the transition from adenoma to carcinoma, increasing the risk of colorectal cancer in this population [9]. If so, this should be reflected in a higher prevalence and an earlier onset of advanced adenomas in FDRs of patients with colorectal cancer than in average-risk individuals (i.e., asymptomatic individuals over 50 y). However, the results of several studies are inconclusive. Some show an increased prevalence of advanced adenoma but are questionable due to small sample size [10] or a retrospective design [11,12], based on registries that could introduce a selection bias because they included patients with hereditary colorectal cancer syndromes, indication of colonoscopy was not specifically ascertained, and information on colonoscopy quality was not documented. On the other hand, few prospective studies that have specifically addressed this issue were underpowered to stratify for familial risk or did not include appropriate average-risk individuals [13–17]. Indeed, there is little evidence supporting the notion that the natural history and prognosis of non-syndromic familial colorectal cancer and sporadic colorectal cancer may differ. Overall, evidence only favors screening at a younger age in familial-risk individuals [5]. In fact, there are no large-scale studies comparing the prevalence of advanced adenomas in asymptomatic familial- and average-risk individuals stratified by number of affected relatives, age and gender. Therefore, clarification on this issue in different familial-risk groups is crucial to guide future screening recommendations for this population.

The current study aimed to compare the neoplastic findings on first screening colonoscopy and the predicted probability of advanced adenoma or cancer according to age and gender between asymptomatic FDR of patients with colorectal cancer and average-risk individuals.

## Methods

### Analysis Plan

The current study was planned in March 2011 as a post-hoc analysis of three prospective studies [18–20]: one study that assessed the perfomance of fecal immunochemical testing in asymptomatic FDR of patients with colorectal cancer [18], one randomized control trial (RCT) that assessed the efficacy of fecal immunochemical testing and colonoscopy as screening methods in asymptomatic FDR of patients with colorectal cancer [19], and one RCT that assessed the efficacy of fecal immunochemical testing and colonoscopy as screening methods in asymptomatic average-risk individuals [20]. Patient recruitment was completed by December 2011 and all colonoscopies were finished by March 2012. The database audit was carried out by EQ and FB and queries were solved between June 2013 and February 2014. Finally, data were analyzed between March and September 2014.

### Study Design and Setting

**Fig 1:**
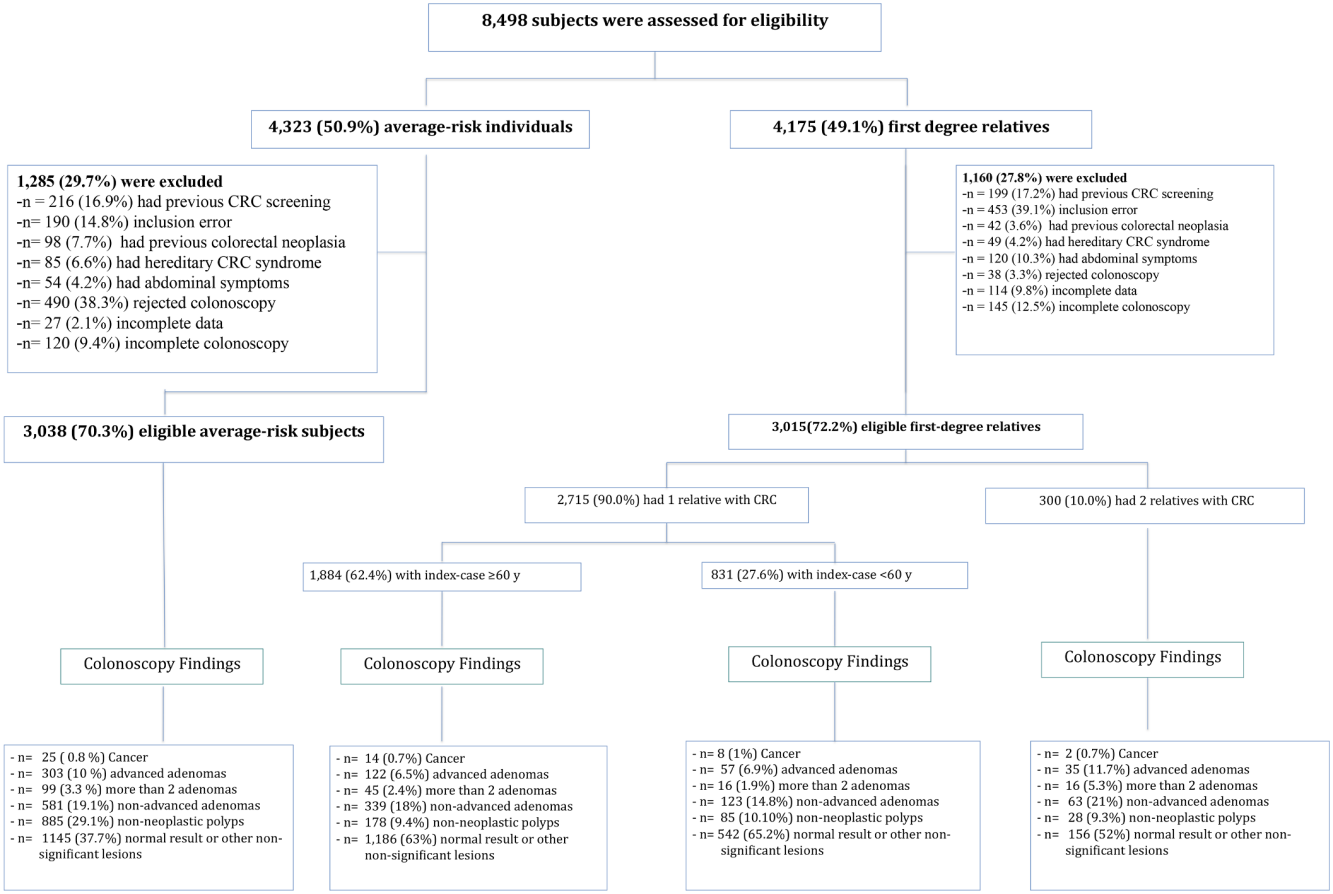
Study enrollment and outcomes. Asymptomatic average-risk individuals and familial-risk individuals undergoing their first lifetime screening colonoscopy were prospectively included in the six participating centers. All had high-quality colonoscopies (good or excellent bowel cleansing and cecal intubation).

The study includes prospectively collected cross-sectional data from asymptomatic whites undergoing their first lifetime screening colonoscopy between January 2006 and March 2012 (Fig 1), attended at six Spanish tertiary hospitals with specific high-risk colorectal cancer clinics responsible for the management of patients with hereditary colorectal cancer syndromes and familial colorectal cancer. In the two studies including asymptomatic familial-risk individuals the recruitment period was from January 2006 to December 2010 [19] and from January 2010 to December 2011 [18], respectively. In the RCT including asymptomatic average-risk individuals the recruitment period was initiated in June 2009, and finished in June 2011.

### Study Sample

An asymptomatic familial-risk group comprising 4,175 asymptomatic FDR of patients with non-syndromic colorectal cancer was created by combining data from multiple sources: 2,322 (55.6%) of the individuals were consecutively included in two prospective trials [18,19] designed to analyze the efficacy of fecal immunochemical testing to detect advanced neoplasia, and the remaining FDR (*n* = 1,853; 44.4%) were recruited from those attending their respective colorectal cancer high-risk outpatient clinics due to family history of colorectal cancer, to complete sample size requirements. These individuals were mainly referred either by primary care physicians or colleagues from the oncology and surgery departments following local referring protocols. Screening recommendations for individuals with family history of colorectal cancer were based on the Spanish Clinical Practice Guideline for colorectal cancer screening [6]. In summary, colonoscopy every 5 y from age 40 (or 10 y before the youngest case in the immediate family) is recommended for individuals with one FDR with colorectal cancer diagnosed before the age of 60 or two or more FDR with colorectal cancer (regardless of age), and colonoscopy every 10 y from age 40 (or 10 y before the youngest case in the immediate family) for individuals with one FDR diagnosed over the age of 60. Baseline and outcome data from FDR included at colorectal cancer high-risk outpatient clinics were prospectively collected in an anonymized database, according to the Spanish personal data protection law.

A second group of asymptomatic individuals with average risk of colorectal cancer, served as a control group, and included 4,323 asymptomatic average-risk individuals (adults 50 to 69 y of age with no family history of colorectal cancer) assigned to the colonoscopy arm in the COLONPREV trial [20], a RCT designed to compare the efficacy of colonoscopy and biennial fecal immunochemical testing for reducing colorectal cancer-related mortality in the average-risk population. The study protocol is available online (www.nejm.org/doi/suppl/10.1056/NEJMoa1108895/suppl_file/nejmoa1108895_appendix.pdf) [20].

FDR of patients with colorectal cancer was determined during an interview by a gastroenterologist and using a questionnaire on demographic data as well as their own medical and family history of cancer at each participant center. Recruitment of individuals in the average-risk cohort has been previously described and included a personal interview about personal and family history of neoplasia performed at the local colorectal cancer screening office [20].

Inclusion criteria for the familial-risk group were the following: age 40–69 or 10 y younger than the youngest case in the family; complete colonoscopy (good or excellent bowel cleansing and cecal intubation), and colorectal cancer confirmed by written medical report. In the average-risk cohort, inclusion criteria were the following: age 50–69 y and no family history of colorectal neoplasia [20].

Exclusion criteria for both groups included the following: personal history of colorectal neoplasia, inflammatory bowel disease, familial history of hereditary colorectal cancer, abdominal symptoms needing further investigation, previous colorectal cancer screening with any technique, severe comorbidity, or refusal to participate.

### Ethics Statement

The Clinical Research Ethics Committee of Hospital Clinic de Barcelona approved the study. The Ethical Committee of Hospital Universitario de Canarias [19] and the Galician Clinical Research Ethics Committee [18] approved the two studies involving familial-risk individuals The ethics committees of the 15 tertiary hospitals participating in the RCT involving average-risk individuals approved the study protocol. All participants belonging to the referred clinical trials [18–20] provided written informed consent and all authors had access to the study data and reviewed and approved the final manuscript. Familial-risk individuals included at the colorectal cancer high-risk outpatient clinics did not provide informed consent as they were waived by the IRB of Hospital Clinic de Barcelona.

### Data Collection

In the familial-risk cohort, age, sex, number of relatives with colorectal cancer, kinship, and index-case age at diagnosis of colorectal cancer were recorded.

In all centers, colonoscopy quality was ensured following the guidelines of the Spanish Gastroenterological Association and the Spanish Society for Digestive Endoscopy [21]. All endoscopists involved in the study had personal experience of more than 200 colonoscopies per year and findings were documented in a standardized report form [20]. The quality of bowel preparation for each colonic segment was categorized as excellent or good versus poor or bad, as previously described [22]. Cases not meeting these requirements or with unexplored cecum were re-scheduled for colonoscopy.

At colonoscopy, the number and size of polyps were recorded. Polyps were classified according to proximal or distal location with respect to the splenic flexure. Adenomas ≥10 mm in size, with tubulovillous architecture, high-grade dysplasia or intramucosal carcinoma were classified as advanced adenomas. The presence of malignant cells observed beyond the *muscularis mucosa* was considered evidence of invasive cancer. Advanced neoplasia was defined as three or more non-advanced adenomas, advanced adenoma or invasive cancer. Patients were classified according to the most advanced lesion.

### Sample Size and Data Analysis

The risk of advanced adenoma and advanced neoplasia according to familial- or average-risk was the main study outcome measure. As the familial-risk population comprised individuals with different neoplastic risk, we estimated the sample size needed to yield sufficient statistical power for significant results with respect to the main outcome measure in the least numerous subgroup, i.e., FDR with two FDR diagnosed with colorectal cancer at any age. Considering a ratio of 10:1 for one versus two relatives affected, an advanced adenoma detection rate of 7.8% in individuals with one FDR and 14.7% in those with two FDR with colorectal cancer, with an alpha error of 0.05 (two-sided) and a beta error of 0.10, the number of individuals having two FDR with colorectal cancer required was 235. Individuals having one family member with colorectal cancer diagnosed at <60 or ≥60 y were analyzed separately, as an age threshold of 60 y in the index-case is considered a colorectal cancer risk factor for their relatives [1,6,12].

Between-group differences in the risk of neoplastic lesions with respect to both overall or colon side-specific colorectal neoplasia detection rates were analyzed by multinomial logistic regression analysis when considering the 4-level categorical variables (normal colonoscopy, non-advanced adenoma, advanced adenoma, and cancer). In these analyses, the most severe colonoscopic finding was represented as an independent category. Binary logistic regression analysis was used when considering another colonoscopic findings included in the previous 4-level categorical variable. All logistic regression analyses were adjusted for age, gender, and center and reported as odds ratios (OR) with 95% confidence intervals (CI) [23].

A logistic regression model was developed to predict the probability of advanced neoplasia according to age, gender and familial groups. The observed frequencies and probabilities predicted from the regression equation were graphically represented stratifying individuals in 10-y subsets (20 to 70 for the affected familial groups, 50 to 70 for the control group) and by gender.

The analysis of resources, based on the number of colonoscopies needed to detect one advanced neoplasia, was performed by inverse marginal probability estimated by binary logistic regression analysis [24], adjusted for age and center. All analyses were performed using SPSS version 15.0 (SPSS Inc., Chicago, IL, United States) and STATA version 13.1 (Stata Corp, TX, US) software. All statistical tests were two-sided, and *p*-values <0.05 were considered statistically significant. STROBE guidelines were followed (S1 Text).

## Results

### Study Population

Overall, 8,498 individuals were assessed for eligibility, of whom 4,175 (49.1%) were assigned to the familial-risk group and 4,323 (50.9%) to the average-risk group. Of these, 1,160 (27.8%) individuals having FDR with colorectal cancer and 1,285 (29.7%) average-risk individuals were excluded (Fig 1) for a total of 3,015 individuals with familial-risk and 3,038 individuals with average-risk included in the analyses.

Demographic data of the familial-risk cohort are shown in Table 1.

**Table 1:** Demographic data.

Category	Average-risk individuals		One FDR with CRC diagnosed at ≥60 y		One FDR with CRC diagnosed at <60 y			Two FDR with CRC diagnosed at any age			Total
**Age group (y), *n* (%)**											
< 40	NA	NA	43	(2.3)	96		(11.6)	9	(3.0)		148 (2.4)
40 to 49	NA	NA	747	(39.6)	315		(37.9)	68	(22.7)		1,130 (18.7)
50 to 59	1,551	(51.1)	666	(35.4)	267		(32.1)	107	(35.7)		2,591 (42.8)
60 to 69	1,487	(48.9)	428	(22.7)	153		(18.4)	116	(38.7)		2,184 (36.1)
**Total**	3,038	(100)	1,884	(100)	831		(100)	300	(100)		6,053 (100)
**Mean age ± SD, y**	59.3 ± 5.5		52.1 ± 8.5		49.7 ± 9.6			55.5 ± 8.9			55.6 ± 8.3
**Gender, *n* (%)**											
Female	1,591	(52.4)	1,107	(58.8)	486		(58.5)	177	(59.0)		3,361 (55.5)
Male	1,447	(47.6)	777	(41.2)	345		(41.5)	123	(41.0)		2,692 (44.5)
**Kinship, *n* (%)** ^a^											
Parents	NA	NA	1,640	(87)	362		(43.6)	232	(77.3)		2,234 (36.9)
Siblings	NA	NA	243	(12.9)	450		(54.2)	233	(77.7)		926 (15.3)
Offspring	NA	NA	1	(0.1)	19		(2.3)	3	(1)		23 (0.4)

FDR = first-degree relatives; CRC = colorectal cancer; NA = not applicable

^a^ The total numbers in each category may exceed the total number of individuals because first-degree relatives may have more than one close relative with CRC.

The number of index-cases was 2,474 (median age 66.2 y). Of these, 1,399 (56.5%) were male and 786 (31.7%) were aged <60 y at diagnosis of colorectal cancer. Siblings with colorectal cancer predominated among individuals having two FDR affected compared to individuals having only one FDR with colorectal cancer (77.7% versus 25.5%, *p* < 0.001) (Table 1). Compared to average risk individuals, mean age of the familial-risk individuals was lower (51.8 ± 9.0 versus 59.3 ± 5.5 y, *p* < 0.001), as was the proportion of men (41.3% versus 47.6%, *p* < 0.001), as shown in Table 1.

### Risk of Advanced Neoplasia

Colonoscopy findings are shown in Fig 1. Compared to average-risk individuals, in individuals having two FDR with colorectal cancer, we observed a higher prevalence and risk of non-advanced adenoma (OR 1.40; 95% CI 1.02 to 1.92, *p* = 0.03), advanced adenoma (OR 2.13; 95% CI 1.42 to 3.19, *p* < 0.001), three or more non-advanced adenomas (OR 2.16, 95% CI 1.21 to 3.84, *p* = 0.01), and advanced neoplasia (OR 1.90; 95% CI 1.36 to 2.66, *p* < 0.001) after adjusting for age, sex, and center (Table 2).

**Table 2:** Risk of colorectal neoplasia in familial- and average-risk groups.

Most advanced lesion	Screening group	n	OR^a^	95% CI	*p*-value
**Non-advanced adenoma**	Average-risk individuals	581	1 ^b^		
	One FDR with CRC diagnosed at ≥ 60 y	339	1.18	0.99 to 1.40	0.05
	One FDR with CRC diagnosed at < 60 y	123	1.07	0.84 to 1.35	0.56
	Two FDR with CRC diagnosed at any age	63	1.40	1.02 to 1.92	0.03
**Advanced adenoma** ^c^	Average-risk individuals	303	1 ^b^		
	One FDR with CRC diagnosed at ≥ 60 y	122	1.09	0.85 to 1.40	0.49
	One FDR with CRC diagnosed at < 60 y	57	1.30	0.93 to 1.81	0.12
	Two FDR with CRC diagnosed at any age	35	2.13	1.42 to 3.19	<0.001
**≥3 Non-advanced adenomas**	Average-risk individuals	99	1 ^b^		
	One FDR with CRC diagnosed at ≥ 60 y	45	0.93	0.62 to 1.38	0.72
	One FDR with CRC diagnosed at < 60 y	16	0.84	0.47 to 1.50	0.56
	Two FDR with CRC diagnosed at any age	16	2.16	1.21 to 3.84	0.01
**Cancer**	Average-risk individuals	25	1 ^b^		
	One FDR with CRC diagnosed at ≥ 60 y	14	1.61	0.80 to 3.25	0.17
	One FDR with CRC diagnosed at < 60 y	8	2.31	0.98 to 5.42	0.05
	Two FDR with CRC diagnosed at any age	2	1.18	0.26 to 5.18	0.82
**Advanced neoplasia** ^d^	Average-risk individuals	427	1 ^b^		
	One FDR with CRC diagnosed at ≥ 60 y	181	1.03	0.83 to 1.27	0.77
	One FDR with CRC diagnosed at < 60 y	81	1.19	0.90 to 1.58	0.20
	Two FDR with CRC diagnosed at any age	53	1.90	1.36 to 2.66	<0.001

OR = odds ratio; CI = confidence interval; FDR = first-degree relatives; CRC = colorectal cancer.

^a^ adjusted for age, gender and participating center.

^b^ Reference category.

^c^ Advanced adenoma included adenoma ≥10 mm in diameter, with tubulovillous architecture or with high-grade dysplasia.

^d^ Advanced neoplasia included advanced adenoma, three or more non-advanced adenomas or CRC. Comparisons with the reference group were carried out using multiple binary logistic regression analysis.

**Fig 2:**
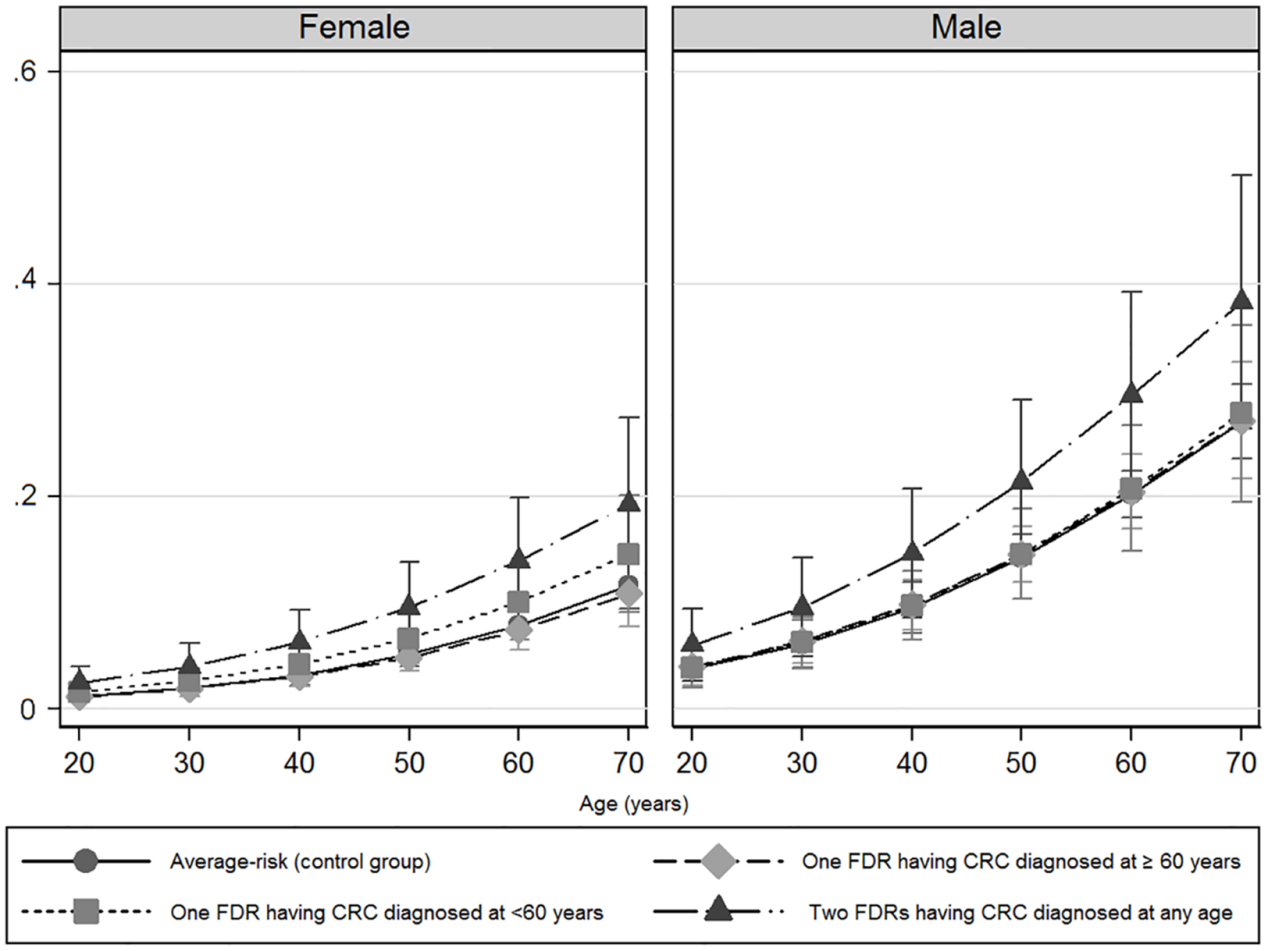
Predicted probabilities of advanced colorectal neoplasia according to age and gender. Women (left) and men (right). A logistic regression model was developed to predict the probability of advanced neoplasia according to age. The observed frequencies and probabilities predicted from the regression equation were graphically represented, stratifying individuals in 10-y subsets (from 20 to 70 y for the affected familial groups, and from 50 to 70 y for the control group). Vertical lines mark the corresponding 95% confidence intervals.

The probability of advanced neoplasia increased with age in both cohorts, as expected (Fig 2).

The prevalence and risk of advanced neoplasia was markedly greater in men than in women in all groups (Table 3) and at each age interval (Fig 2).

**Table 3:** Risk of colorectal neoplasia according to gender in familial- and average-risk groups.

Most advanced lesion	Screening group	*n*	Men	Women	OR^a^	95% CI	*p*-value
**Non-advanced adenoma**	Average-risk individuals	581	326	255	1.52	1.26 to 1.82	<0.001
	One FDR with CRC diagnosed at ≥60 y	339	162	177	1.42	1.12 to 1.81	0.004
	One FDR with CRC diagnosed at <60 y	123	55	68	1.23	0.87 to 1.82	0.306
	Two FDR with CRC diagnosed at any age	63	24	39	0.91	0.50 to 1.64	0.763
**Advanced adenoma** ^b^	Average-risk individuals	303	206	97	2.66	2.06 to 3.44	<0.001
	One FDR with CRC diagnosed at ≥60 y	122	77	45	2.65	1.80 to 3.88	<0.001
	One FDR with CRC diagnosed at <60 y	57	32	25	2.02	1.16 to 3.51	0.013
	Two FDR with CRC diagnosed at any age	35	19	16	1.88	0.90 to 3.91	0.088
**≥3 Non-advanced Adenomas**	Average-risk individuals	99	74	25	3.33	2.10 to 5.28	<0.001
	One FDR with CRC diagnosed at ≥60 y	45	32	13	3.73	1.94 to 7.18	<0.001
	One FDR with CRC diagnosed at <60 y	16	13	3	7.24	2.01 to 26.09	0.002
	Two FDR with CRC at any age	16	11	5	3.59	1.20 to 10.79	0.022
**Cancer**	Average-risk individuals	25	17	8	2.42	1.03 to 5.66	0.041
	One FDR with CRC diagnosed at ≥60 y	14	10	4	3.78	1.16 to 12.24	0.027
	One FDR with CRC diagnosed at <60 y	8	4	4	1.42	0.34 to 5.84	0.623
	Two FDR with CRC diagnosed at any age	2	1	1	2.08	0.11 to 39.08	0.622
**Advanced neoplasia** ^c^	Average-risk individuals	427	297	130	2.99	2.39 to 3.74	<0.001
	One FDR with CRC diagnosed at ≥60 y	181	119	62	3.16	2.28 to 4.37	<0.001
	One FDR with CRC diagnosed at <60 y	81	49	32	2.57	1.59 to 4.17	<0.001
	Two FDR with CRC diagnosed at any age	53	31	22	2.50	1.34 to 4.65	0.004

CI = confidence interval; FDR = first-degree relatives; CRC = colorectal cancer; OR = odds ratio.

^a^ Odds ratio compared men versus women, adjusted for age and participating center.

^b^ Advanced adenoma included adenoma ≥10 mm in diameter, with tubulovillous architecture or with high-grade dysplasia.

^c^ Advanced neoplasia included advanced adenoma, three or more non-advanced adenomas or CRC. Comparisons with the reference group were carried out using multiple binary logistic regression analysis.

Men and women having one FDR with colorectal cancer consistently showed a similar probability of advanced neoplasia than average-risk men and women, respectively (Fig 2). Interestingly, after the age of 50 y, advanced neoplasia was over 2-fold higher (OR 2.50; 95% CI 1.36–2.66, *p* < 0.001) and developed at least 10 y earlier in men compared to women in the subgroup of individuals having two FDR with colorectal cancer, (Fig 2 and Table 4).

**Table 4:** Advanced adenomas^a^ and advanced neoplasia^b^ found at colonoscopy according to age and gender.

Age-group (y)		Average-risk individuals		One FDR with CRC diagnosed at ≥60 y		One FDR with CRC diagnosed at <60 y		Two FDRs with CRC diagnosed at any age	
	Category	Men	Women	Men	Women	Men	Women	Men	Women
20–29 (*n* = 19)	Advanced adenoma, *n* (%)	N/A	N/A	0/1 (0)	0/2 (0)	0/7 (0)	0/8 (0)	0/1 (0)	0/0 (0)
	Advanced neoplasia, *n* (%)	N/A	N/A	0/1 (0)	0/2 (0)	0/7 (0)	0/8 (0)	0/1 (0)	0/0 (0)
30–39 (*n* = 132)	Advanced adenoma, *n* (%)	N/A	N/A	1/17 (5.9)	0/26 (0)	2/46 (4.3)	1/35 (2.9)	0/4 (0)	1/4 (25)
	Advanced neoplasia, *n* (%)	N/A	N/A	2/17 (11.8)	0/26 (0)	2/46 (4.3)	2/35 (5.7)	1/4 (25)	1/4 (25)
40–49 (*n* = 1,130)	Advanced adenoma, *n* (%)	N/A	N/A	24/326 (7.4)	13/424 (3.1)	4/124 (3.2)	6/192 (3.1)	4/32 (12.5)	1/36 (2.8)
	Advanced neoplasia, *n* (%)	N/A	N/A	36/326 (11.0)	13/424 (3.1)	7/124 (5.6)	7/192 (3.6)	4/32 (12.5)	4/36 (11.1)
50–59 (*n* = 2,591)	Advanced adenoma, *n* (%)	90/756 (11.9)	47/795 (5.9)	30/262 (11.5)	21/409 (5.1)	14/99 (14.1)	13/171 (7.6)	8/44 (18.2)	6/65 (9.2)
	Advanced neoplasia, *n* (%)	124/756 (16.4)	65/795 (8.2)	43/262 (16.4)	31/409 (7.6)	22/99 (22)	15/171 (8.8)	14/44 (31.8)	7/65 (10.8)
60–69 (*n* = 2,184)	Advanced adenoma, *n* (%)	116/691 (16.8)	50/796 (6.3)	22/174 (12.6)	11/255 (4.3)	12/70 (17.1)	6/84 (7.1)	8/45 (17.8)	8/72 (11.4)
	Advanced neoplasia, *n* (%)	173/691 (25.0)	65/796 (8.2)	38/174 (21.8)	18/255 (7.1)	18/70 (25.7)	9/84 (10.7)	13/45 (28.9)	10 /72(13.9)

FDR = asymptomatic first-degree relatives aged 40 to 69 or 10 y earlier than the youngest case with CRC in the family; CRC = colorectal cancer

^a^ Advanced adenoma included adenoma ≥10 mm in diameter, with tubulovillous architecture or with high-grade dysplasia.

^b^ Advanced neoplasia included advanced adenoma, three or more non-advanced adenomas, or CRC

Individuals having two FDR with colorectal cancer showed a significantly higher prevalence and risk of advanced adenomas than average-risk individuals both in distal (OR 2.08; 95% CI 1.35 to 3.19, *p* = 0.001) and proximal colon (OR 1.92; 95% CI 1.08 to 3.40, *p* = 0.026) (Table 5).

**Table 5:** Risk of advanced adenoma^a^ stratified by location of lesion^b^.

Category	Screening group	Individuals with advanced adenoma, *n*	OR^c^	95% CI	*p*-value
**Overall**	Average-risk individuals (*n* = 3,038)	303	1^d^		
	One FDR having CRC diagnosed at ≥60 y (*n* = 1,884)	122	1.03	0.81 to 1.32	0.774
	One FDR having CRC diagnosed at <60 y (*n* = 831)	57	1.24	0.89 to 1.72	0.195
	Two FDRs having CRC diagnosed at any age (*n* = 300)	35	1.86	1.26 to 2.76	0.002
**Distal**	Average-risk individuals (*n* = 3,038)	231	1^d^		
	One FDR having CRC diagnosed at ≥60 y (*n* = 1,884)	99	1.35	0.86 to 1.49	0.360
	One FDR having CRC diagnosed at <60 y (*n* = 831)	45	1.33	0.93 to 1.92	0.117
	Two FDRs having CRC diagnosed at any age (*n* = 300)	29	2.08	1.35 to 3.19	0.001
**Proximal**	Average-risk individuals (*n* = 3,038)	130	1^d^		
	One FDR having CRC diagnosed at ≥60 y (*n* = 1,884)	41	0.89	0.60 to 1.31	0.564
	One FDR having CRC diagnosed at <60 y (*n* = 831)	18	1.00	0.59 to 1.72	0.988
	Two FDRs having CRC diagnosed at any age (*n* = 300)	15	1.92	1.08 to 3.40	0.026

FDR = asymptomatic first-degree relatives aged 40 to 69 or 10 y earlier than the youngest case with CRC in the family; CRC = colorectal cancer; CI = confidence interval.

^a^ Advanced adenoma included adenomas >10 mm in diameter, with tubulovillous architecture or with high-degree dysplasia.

^b^ Neoplasm location was established with respect to the splenic flexure; the total number of individuals with proximal and distal lesions may exceed the total number of individuals because individuals could have lesions in both locations.

^c^Adjusted by age, gender, and center.

^d^ Reference category.

No differences in the prevalence and risk of advanced adenomas categorized by location were observed for individuals having only one FDR with colorectal cancer compared to average-risk individuals.

### Analysis of Resources

The number of colonoscopies needed to detect one advanced neoplasia was 7 (95% CI 5.4 to 10) in individuals having two FDR with colorectal cancer, 10.6 (95% CI 9.2 to 12.6) in individuals having only one FDR with colorectal cancer diagnosed at <60 y, 9 (95% CI 7.2 to 11.6) in individuals having one FDR diagnosed at >=60 y, and 11.6 (95% CI 10.4 to 13) in average-risk individuals. Approximately 2-fold fewer colonoscopies were needed to detect one advanced neoplasia in men than in women in all groups (Table 6).

**Table 6:** Number of colonoscopies needed to detect one advanced adenoma^a^ and one advanced neoplasia^b^ according to familial risk.

Category	Average-risk individuals *n* (95% CI)	One FDR with CRC diagnosed at <60 y *n* (95% CI)	One FDR with CRC diagnosed at ≥60 y *n* (95% CI)	Two FDRs with CRC diagnosed at any age *n* (95% CI)
**Advanced adenoma**				
Men	7.9 (7.0 to 9.1)	7.6 (6.3 to 9.7)	7.2 (5.5 to 10.5)	5.1 (3.7 to 8.4)
Women	19.9 (16.6 to 24.8)	19.3 (15.0 to 27.0)	13.6 (9.9 to 21.8)	9.4 (6.5 to17.3)
Global	12.0 (10.8 to 13.5)	11.6 (9.9 to 14.0)	9.8 (7.8 to 13.0)	6.9 (5.3 to 9.8)
**Advanced neoplasia**				
Men	7.8 (7.0 to 9.0)	7.2 (6.0 to 8.8)	6.8 (5.2 to 9.8)	5.4 (4.0 to 8.8)
Women	18.8 (15.8 to 23.2)	17.8 (14.0 to 24.6)	11.8 (8.8 to 18.2)	9.4 (6.4 to16.8)
Global	11.6 (10.4 to 13)	10.6 (9.2 to 12.6)	9.0 (7.2 to 11.6)	7.0 (5.4 to 10.0)

FDR = asymptomatic first-degree relatives aged 40 to 69 or 10 y younger than the youngest case with CRC in the family; CRC = colorectal cancer; CI = confidence interval.

^a^Advanced adenoma included adenomas >10 mm in diameter, with tubulovillous architecture or with high-degree dysplasia.

^b^Advanced neoplasia included advanced adenoma, three or more non-advanced adenomas, or CRC.

## Discussion

The current study adds new insight on the risk and probability of colorectal neoplasia in the familial-risk population. Men and women having two FDR with colorectal cancer showed a significantly greater risk of advanced adenomas and advanced neoplasia than those with average-risk. In contrast, men and women having one FDR with colorectal cancer showed a similar risk of these lesions to average-risk individuals, regardless of index-case age at diagnosis of colorectal cancer. The risk of advanced neoplasia was almost 3-fold higher and appeared at least 10 y earlier in men than in women in both the familial- and average-risk groups.

Advanced adenoma and early colorectal cancer are surrogate endpoints of colorectal cancer screening because the detection and treatment of these lesions is associated with a significant reduction of colorectal cancer incidence and mortality [25,26]. Based on this principle, and on population-based studies reporting that FDR of patients with colorectal cancer have a higher relative risk of developing the disease than the average-risk population [12,27,28], current guidelines recommend screening colonoscopy every 5 [3,4,6–8,29] or 10 [3] y, depending on whether colorectal cancer in the index-case is diagnosed at <60 or ≥60 y. However, there is no clear evidence that the natural history of pre-cancerous lesions and cancer differs between familial- and average-risk populations. In this regard, the risk of adenoma recurrence is more related to the characteristics of the neoplasia at baseline colonoscopy and to demographic data (age and gender) than to family history, suggesting that screening intervals in individuals with familial colorectal cancer could be extended beyond 5 y, as most guidelines recommend [30,31].

Previous case-control studies have shown contradictory results with respect to adenoma prevalence in familial colorectal cancer. At least two studies [32,33] have reported a similar prevalence of adenomas in individuals having one FDR with colorectal cancer compared with average-risk individuals. In contrast, other studies have shown an increased prevalence of advanced adenomas in FDR of patients with colorectal cancer compared with average-risk individuals [11–13,34,35]. However, some of these studies had important methodological flaws: first, the control group was small in most of them [13,32,33,35] and frequently inappropriate, including autopsies [10], symptomatic patients [35], or volunteers paying for screening colonoscopy [13]. Second, the small sample size did not allow stratification according to the number of FDR affected [13,32,33,35]. Third, their retrospective design did not allow for ascertaining the indication for colonoscopy and exclusion of patients with hereditary colorectal cancer syndromes [11,12]. Finally, high-quality colonoscopy was not specifically assessed in many studies [10–12,32,33].

Our study apparently solved these drawbacks, corroborating that among relatives of patients with colorectal cancer, only those with two FDR affected showed a marked increase in the prevalence and risk of both adenomas and cancer compared with average-risk individuals in both the proximal and the distal colon. However, the risk of advanced neoplasia in individuals having only one FDR with colorectal cancer diagnosed before or after the age of 60 y was similar to that of average-risk individuals. These findings are in line with the results of a nested study performed within the randomized controlled Prostate, Lung, Colorectal, and Ovarian (PLCO) cancer-screening trial of flexible sigmoidoscopy versus usual care, showing that men and women having two FDR with colorectal cancer had a 2-fold increased risk of incident colorectal cancer, whereas those with one FDR affected were not associated with an increased risk in colorectal cancer incidence or mortality, regardless of their age at the time of diagnosis [36].

Interestingly, we observed that individuals with two FDR had also an increased risk of both non-advanced and advanced-adenomas compared with average-risk individuals. In addition, men showed an almost 3-fold higher risk of advanced neoplasia than women in all groups, and advanced neoplasia appeared at least 10 y earlier in men than in women. It is interesting to note that the number of colonoscopies needed to detect one advanced neoplasia was 2-fold higher in women than in men at all ages in both cohorts. Taken together, our results support the notion that screening colonoscopy may be delayed at least 10 y in women having one or even two FDR with colorectal cancer, as has been shown for women in the average-risk population [37–39].

Our study has several strengths. First, participants were recruited with strict selection criteria regarding colonoscopy quality and inclusion age. Only individuals with a first lifetime complete colonoscopy were eligible and an upper age limit of 69 y was established to minimize the effect of age on the risk of advanced neoplasia. Second, colonoscopies were performed by the same endoscopists who applied the same quality criteria in both cohorts. Third, to our knowledge, this is the largest study to compare the risk of advanced neoplasia between asymptomatic FDR of patients with colorectal cancer and average-risk individuals, thus allowing an accurate estimation of this parameter according to the number of close relatives with colorectal cancer, gender, and index-case age at diagnosis of the disease.

The study also has certain limitations. First, although average-risk individuals were consecutively included in the COLONPREV study [20] this was not the case in the familial-risk group. Fifty-five percent of the FDR were consecutively included in two prospective studies [18,19], whereas the remaining FDR were not. However, demographic characteristics and risk of advanced neoplasia were similar in FDR regardless of whether they were included consecutively or not (S1 Table). This potential bias was also minimized by performing a logistic regression analysis, controlling for confounding factors. Second, due to the study design, there was a substantial difference in age between individuals with family history of colorectal cancer and average risk individuals. Both logistic regression analyses and stratification by age suggested that age was not a main confounding factor in this study (S2 Table). However we cannot exclude an immortal time bias in the study. The risk of bias is built into the study design in a way that cannot be compensated by including an interaction test for age. Third, other confounding factors such as nonsteroidal anti-inflammatory drugs, acetylsalicylic acid, smoking, obesity, and diet were not recorded, and they could conceivably have influenced the results. Fourth, unfortunately, the prevalence of serrated polyps was not recorded because at the time the present study was initiated, classification of serrated polyps was still under debate. In fact, only recently clinical guidelines recommend colonoscopy surveillance for individuals with serrated polyps [1]. Fifth, colorectal cancer family history was obtained by interview and therefore could be underreported [40]. Additionally, the effect of the number of colorectal cancers in relation to the family size could not be analyzed. Sixth, although adenoma detection rate is widely used as a colonoscopy quality indicator, unfortunately this information was not available. Since the centers that provided individuals in the average-risk group (COLONPREV) and the ones that recruited individuals in the familial-risk cohort are virtually the same, we do not expect differences in colonoscopy quality between the two cohorts. Finally, some of the individuals included may have been members of families with Lynch syndrome, since we did not systematically exclude DNA mismatch repair deficiency in all cases with colorectal cancer. However, the overall results should not be greatly affected since this possibility would only involve a small number of individuals.

Our findings suggest that screening guidelines for the management of familial colorectal cancer, if not adjusted for the number of relatives affected and sex, may substantially overestimate the prevalence of advanced neoplasia, particularly in men and women having one FDR with colorectal cancer and in women having two FDR with colorectal cancer diagnosed before the age of 50. In fact, the same screening strategy as that for average-risk individuals could be recommended to men and women having only one FDR with colorectal cancer, but starting at the age of 40 or 45 y, in line with the results of previous studies [5,30], thus avoiding overuse of screening colonoscopy. In support of this recommendation, there are two recent prospective studies demonstrating that fecal immunochemical testing is as effective as colonoscopy to detect advanced neoplasia in colorectal cancer associated with familial risk [19,41]. In contrast, the higher prevalence and earlier presentation of advanced neoplasia in men having two FDR with colorectal cancer suggest that men have higher genetic penetrance, thus supporting screening colonoscopy from the age of 40, whereas it could be delayed until the age of 50 or older in women with two FDR.

In conclusion, our study demonstrates that the risk and predicted probability of advanced neoplasia are markedly increased in individuals having two FDR with colorectal cancer compared to average-risk individuals, but not in individuals having only one FDR with colorectal cancer, regardless of when they were diagnosed. Our results suggest that men having two FDR with colorectal cancer may benefit from an early screening colonoscopy (i.e., 40 y old or 10 y before the youngest case in the immediate family), whereas individuals having only one FDR with colorectal cancer could be recommended to undergo the same screening strategy as the average-risk population.

## Supporting Information

S1 Table Demographic data and prevalence of advanced colorectal neoplasia in first-degree relatives according to whether they were included consecutively or not. (DOCX)

S2 Table Risk of colorectal neoplasia in first-degree relatives stratified by age. (DOCX)

S1 Text STROBE checklist. (DOC)

## Abbreviations

CI: confidence intervals
FDR: First-degree relatives
OR: odds ratios
