# Supplementary material for: Risk of Advanced Neoplasia in First-Degree Relatives with Colorectal Cancer: A Large Multicenter Cross-Sectional Study
Source: PLoS Med. 2016 May 3;13(5):e1002008. doi: 10.1371/journal.pmed.1002008 (PMC4854417; doi:10.1371/journal.pmed.1002008)
Supplement: S1 Table — (DOCX) [file pmed.1002008.s001.docx]

**S1 Table**. Demographic data and prevalence of advanced colorectal neoplasia in first-degree relatives according to whether they were included consecutively or not.

| **Category** | **FDR included consecutively**  **n (%)** | **FDR not included consecutively**  **n (%)** |
| --- | --- | --- |
| **Age group (years), *n (%)*** |  |  |
| < 40 | 54 (3.4) | 94 (6.6) |
| 40 to 49 | 689 (43.3) | 441 (31.0) |
| 50 to 59 | 528 (33.2) | 512 (36.0) |
| 60 to 69 | 321 (20.2) | 376 (26.4) |
| Total | 1,592 (100) | 1,423 (100) |
| **Mean age ± SD, years** | 51.1 ± 8.6 | 52.5 ± 9.3 |
| **Gender, n (%)** |  |  |
| Female | 933 (52.7) | 837 (47.3) |
| Male | 659 (52.9) | 586 (47.10) |
| **Kinship, n (%)^a^** |  |  |
| Parents | 487 (30.6) | 439 (30.8) |
| Siblings | 1,164 (73.1) | 1,070 (75.2) |
| Offspring | 17 (1.1) | 6 (0.4) |
| **Advanced adenoma^c^** | 181 (11.4) | 134 (9.2) |
| **Colorectal cancer** | 11 (0.7) | 13 (0.9) |
| **Advanced neoplasia^d^** | 299 (11.7) | 443 (12.6) |

^a^ The total numbers in each category may exceed the total number of individuals because first-degree relatives may have more than one close relative with CRC.

^b^ Non applicable

^c^Advanced adenoma included adenoma ≥ 10 mm in diameter, with tubulovillous architecture or with high-grade dysplasia.

^d^Advanced neoplasia included advanced adenoma, ≥3 non-advanced adenomas or CRC.
