# Supplementary material for: Risk of Advanced Neoplasia in First-Degree Relatives with Colorectal Cancer: A Large Multicenter Cross-Sectional Study
Source: PLoS Med. 2016 May 3;13(5):e1002008. doi: 10.1371/journal.pmed.1002008 (PMC4854417; doi:10.1371/journal.pmed.1002008)
Supplement: S2 Table — (DOCX) [file pmed.1002008.s002.docx]

**S2 Table.** **Risk of colorectal neoplasia in first-degree relatives stratified by age.**

| **Most advanced lesion** | **Age group (years)** | | **Screening group** | | **OR** | | | **95%CI** | | | **P** | | |
| --- | --- | --- | --- | --- | --- | --- | --- | --- | --- | --- | --- | --- | --- |
| Non-advanced adenoma | <60 | | 1 FDR >60  1 FDR <60  2 FDR | | 0.94  0.73  1.16 | | | 0.77 to 1.15  0.56 to 0.96  0.77 to 1.73 | | | 1.15  0.96  0.484 | | |
|  | >60 | | 1 FDR >60  1 FDR <60  2 FDR | | 1.06  1.23  1.34 | | | 0.79 to 1.42  0.79 to 1.93  0.82 to 2.20 | | | 0.681  0.349  0.237 | | |
| Advanced adenoma ^a^ | | <60 | | 1 FDR >60  1 FDR <60  2 FDR | | 0.88  0.88  1.76 | 0.66 to 1.18  0.60 to 1.30  1.07 to 2.89 | | | 0.397  0.532  0.025 | | |  |
|  |  | >60 | | 1 FDR >60  1 FDR <60  2 FDR | | 0.89  1.39  1.55 | 0.58 to 1.36  0.79 to 2.43  0.82 to 2.93 | | 0.587  0.257  0.178 | | | |  |
| ≥ 3 Non-advanced adenoma | | <60 | | 1 FDR >60  1 FDR <60  2 FDR | | 0.77  0.49  1.65 | 0.32 to 1.42  0.24 to 1.01  0.81 to 3.36 | | 0.264  0.051  0.171 | | |  |  |
|  |  | >60 | | 1 FDR >60  1 FDR <60  2 FDR | | 0.67  1.22  1.65 | 0.32 to 1.42  0.50 to 3.01  0.32 to 1.42 | | 0.300  0.662  0.300 | | |  |  |
| Colorectal  Cancer | | <60 | | 1 FDR >60  1 FDR <60  2 FDR | | 0.47  0.91  0.82 | 0.15 to 1.46  0.29 to 2.88  0.10 to 6.70 | | 0.191  0.870  0.854 | | |  |  |
|  |  | >60 | | 1 FDR >60  1 FDR <60  2 FDR | | 2.17  2.12  1.01 | 0.91 to 5.22  0.58 to 7.80  0.13 to 8.12 | | 0.083  0.256  0.988 | | |  |  |
| Advanced  Neoplasia ^b^ | | <60 | | 1 FDR >60  1 FDR <60  2 FDR | | 0.82  0.71  1.86 | 0.64 to 1.07  0.50 to 1.01  1.20 to 2.88 | | 0.144  0.054  0.005 | | |  |  |
|  |  | >60 | | 1 FDR >60  1 FDR <60  2 FDR | | 0.85  1.50  1.82 | 0.57 to 1.25  0.89 to 2.53  1.02 to 3.25 | | 0.408  0.128  0.044 | | |  |  |

OR Logistic Regression

CI = confidence interval; FDR = first-degree relatives; CRC = colorectal cancer; Reference category: average risk individuals

^a^ Advanced adenoma included adenoma ≥ 10 mm in diameter, with tubulovillous architecture or with high-grade dysplasia.

^b^ Advanced neoplasia included advanced adenoma, ≥3 non-advanced adenomas or CRC. Comparisons with the reference group were carried out using multiple binary logistic regression analysis.
