# Supplementary material for: Risk of Advanced Neoplasia in First-Degree Relatives with Colorectal Cancer: A Large Multicenter Cross-Sectional Study
Source: PLoS Med. 2016 May 3;13(5):e1002008. doi: 10.1371/journal.pmed.1002008 (PMC4854417; doi:10.1371/journal.pmed.1002008)
Supplement: S1 Text — (DOC) [file pmed.1002008.s003.doc]

**Risk of Advanced Neoplasia in First-Degree Relatives with Colorectal Cancer: a large multicenter cross-sectional study**

STROBE Statement—checklist of items that should be included in reports of observational studies

|  | Item No | Recommendation |
| --- | --- | --- |
| **Title and abstract** | 1 | (*a*) Indicate the study’s design with a commonly used term in the title or the abstract  Risk of Advanced Neoplasia in First-Degree Relatives with Colorectal Cancer: a large multicenter cross-sectional study |
|  |  | (*b*) Provide in the abstract an informative and balanced summary of what was done and what was found  **Background:** First-degree relatives (FDR) of patients with colorectal cancer have a higher risk of developing colorectal cancer than the general population. For this reason, screening guidelines recommend colonoscopy every 5 or 10 years, starting at the age of 40, depending on whether colorectal cancer in the index-case is diagnosed at <60 or ≥60 years, respectively. However, studies on the risk of neoplastic lesions are inconclusive. The aim of this study was to determine the risk of advanced neoplasia (≥3 non-advanced adenomas, advanced adenoma or invasive cancer) in FDR of patients with colorectal cancer compared to average-risk individuals (i.e. asymptomatic adults 50 to 69 years of age with no family history of colorectal cancer), using two prospective cohorts.  **Methods and Findings:** This cross-sectional study involved asymptomatic 3,015 first-degree relatives of patients with colorectal cancer and 3,038 average-risk individuals undergoing their first lifetime screening colonoscopy between 2006 and 2012. The familial-risk cohort was stratified as 1-FDR with colorectal cancer diagnosed at ≥60 years (n=1,884) or at <60 years (n=831) and 2-FDR with colorectal cancer at any age (n=300). Multiple logistic regression analysis was used for between-group comparisons after adjusting for potential confounders. Advanced neoplasia was significantly more prevalent in individuals having 2-FDR with colorectal cancer compared to average-risk individuals (OR 1.90; 95% CI 1.36-2.66, P<0.001), but not in those having 1-FDR with colorectal cancer diagnosed at ≥60 years (OR 1.03; 95% CI 0.83-1.27, P=0.77) and <60 years (OR 1.19; 95% CI 0.90-1.58, P=0.20). After the age of 50 years, men developed advanced neoplasia over two-fold more frequently than women and advanced neoplasia appeared at least ten years earlier. Two-fold fewer colonoscopies were required to detect one advanced neoplasia in men than in women.  Major limitations of this study were first that although average-risk individuals were consecutively included in a randomized control trial, this was not the case for all individuals in the familial-risk cohort; and second, the difference in age between the average-risk and familial-risk cohorts.  **Conclusions:** Individuals having 2-FDR with colorectal cancer showed an increased risk of advanced neoplasia compared to those having 1-FDR with colorectal cancer or average-risk individuals. Men had over two-fold higher risk of advanced neoplasia than women, independently of family history. These data suggest that screening colonoscopy guidelines should be revised in the familial risk population. |
| Introduction | | |
| Background/rationale | 2 | Explain the scientific background and rationale for the investigation being reported  **Introduction:** Clinical guidelines recommend more intensive surveillance in first-degree relatives (FDR) of patients with colorectal cancer than in average-risk individuals, as their risk of developing colorectal cancer is two to four-fold higher . Screening in this population is recommended as from the age of 40 or ten years before the youngest case in the immediate family, since the disease tends to develop about ten years earlier in FDR than in the general population . Colonoscopy every five or ten years, depending on the number of relatives affected and age at cancer diagnosis, is the predominant screening strategy for these individuals, as it is the most effective procedure to detect and remove advanced adenomas. However, this recommendation is not supported by prospective studies.  Low-penetrance genetic alterations may favor earlier development of advanced adenomas or accelerate the transition from adenoma to carcinoma, increasing the risk of colorectal cancer in this population . If so, this should be reflected in a higher prevalence and an earlier onset of advanced adenomas in FDRs of patients with colorectal cancer than in average-risk individuals (i.e. asymptomatic individuals over 50 years).  However, the results of several studies are inconclusive. Some show an increased prevalence of advanced adenoma but are questionable due to small sample size or a retrospective design , based on registries that could introduce a selection bias as they included patients with hereditary colorectal cancer syndromes, indication of colonoscopy was not specifically ascertained and information on colonoscopy quality was not documented. On the other hand, few prospective studies that have specifically addressed this issue were underpowered to stratify for familial risk or did not include appropriate average-risk individuals . Indeed, there is little evidence supporting the notion that the natural history and prognosis of non-syndromic familial colorectal cancer and sporadic colorectal cancer may differ. Overall, evidence only favors screening at a younger age in familial risk individuals , In fact, there are no large-scale studies comparing the prevalence of advanced adenomas in asymptomatic familial- and average-risk individuals stratified by number of affected relatives, age and gender. Therefore, clarification on this issue in different familial risk-groups is crucial to guide future screening recommendations for this population. |
| Objectives | 3 | State specific objectives, including any prespecified hypotheses  The current study aimed to compare the neoplastic findings on first screening colonoscopy and the predicted probability of advanced adenoma or cancer according to age and gender between asymptomatic FDR of patients with colorectal cancer and average-risk individuals. |
| Methods | | |
| Study design | 4 | Present key elements of study design early in the paper  **Study design:** The study included two cohorts of asymptomatic Caucasians undergoing their first lifetime screening colonoscopy between January 2006 and March 2012 (Figure 1), attended at six Spanish tertiary hospitals with specific high-risk colorectal cancer clinics responsible for the management of patients with hereditary colorectal cancer syndromes and familial colorectal cancer. Baseline and outcome data were prospectively collected.  The first cohort comprised 4,175 asymptomatic FDR of patients with non-syndromic colorectal cancer, of whom 2,322 (55.6%) were consecutively included in two prospective trials designed to analyze the efficacy of fecal immunochemical testing to detect advanced neoplasia. The remaining FDR (n=1,853) were recruited from those attending their respective colorectal cancer high-risk outpatient clinics due to family history of colorectal cancer. These individuals were mainly referred either by primary care physicians or colleagues from the oncology and surgery departments following local referring protocols. Screening recommendations for individuals with family history of colorectal cancer were based on the Spanish Clinical Practice Guideline for colorectal cancer screening . In summary, colonoscopy every 5 years from age 40 (or ten years before the youngest case in the immediate family) is recommended for individuals with 1 FDR with CRC diagnosed before the age of 60 or >2 FDR with CRC (regardless of age), and colonoscopy every 10 years from age 40 (or ten years before the youngest case in the immediate family) for individuals with 1 FDR diagnosed over the age of 60.  The second cohort, who served as controls, included 4,323 asymptomatic average-risk individuals (adults 50 to 69 years of age with no family history of colorectal cancer) assigned to the colonoscopy arm in the COLONPREV trial , a randomized control trial designed to compare the efficacy of colonoscopy and biennial fecal immunochemical testing for reducing colorectal cancer-related mortality in the average-risk population. The study protocol is available online . |
| Settingdata | 5 | Describe the setting, locations, and relevant dates, including periods of recruitment, exposure, follow-up, and data collection  Setting, locations and dates are described in the study design.  **Data collection:** In the familial-risk cohort, age, sex, number of relatives with colorectal cancer, kinship and index-case age at diagnosis of colorectal cancer were recorded.  In all centers, colonoscopy quality was ensured following the guidelines of the Spanish Gastroenterological Association and the Spanish Society for Digestive Endoscopy . All endoscopists involved in the study had personal experience of more than 200 colonoscopies per year and findings were documented in a standardized report form . The quality of bowel preparation for each colonic segment was categorized as excellent or good versus poor or bad, as previously described . Cases not meeting these requirements or with unexplored cecum were re-scheduled for colonoscopy.  At colonoscopy, the number and size of polyps were recorded. Polyps were classified according to proximal or distal location with respect to the splenic flexure. Adenomas ≥10 mm in size, with tubulovillous architecture, high-grade dysplasia or intramucosal carcinoma were classified as advanced adenomas. The presence of malignant cells observed beyond the muscularis mucosa was considered evidence of invasive cancer. Advanced neoplasia was defined as ≥3 non-advanced adenomas, advanced adenoma or invasive cancer. Patients were classified according to the most advanced lesion. |
| Participants | 6 | (*a*) *Cohort study*—Give the eligibility criteria, and the sources and methods of selection of participants. Describe methods of follow-up  *Case-control study*—Give the eligibility criteria, and the sources and methods of case ascertainment and control selection. Give the rationale for the choice of cases and controls  *Cross-sectional study*—Give the eligibility criteria, and the sources and methods of selection of participants  **Study population:** FDR of patients with colorectal cancer were interviewed by a gastroenterologist and completed a questionnaire on demographic data as well as their own medical and family history of cancer. Recruitment of individuals in the average-risk cohort has been previously described and included a personal interview about personal and family history of neoplasia performed at the local colorectal cancer screening office .  Inclusion criteria for the familial-risk cohort were: age 40-69 or ten years less than the youngest case in the family; complete colonoscopy (good or excellent bowel cleansing and cecal intubation) and colorectal cancer confirmed by written medical report. In the control cohort, inclusion criteria were: age 50-69 years; no family history of colorectal neoplasia .  Exclusion criteria for both cohorts included: personal history of colorectal neoplasia, inflammatory bowel disease; familial history of hereditary colorectal cancer; abdominal symptoms needing further investigation; previous colorectal cancer screening with any technique; severe comorbidity; or refusal to participate. |
|  |  | (*b*) *Cohort study*—For matched studies, give matching criteria and number of exposed and unexposed  *Case-control study*—For matched studies, give matching criteria and the number of controls per case |
| Variables | 7 | Clearly define all outcomes, exposures, predictors, potential confounders, and effect modifiers. Give diagnostic criteria, if applicable  All outcomes are defined in the Data collection section. |
| Data sources/ measurement | 8* | For each variable of interest, give sources of data and details of methods of assessment (measurement). Describe comparability of assessment methods if there is more than one group  Source of data and methodology are specified in the Data collection section. |
| Bias | 9 | Describe any efforts to address potential sources of bias  All logistic regression analyses were adjusted for age, gender and center and reported as odds ratios (OR) with 95% confidence intervals (CI).  Potential source of biases is explained in the Statistical and Discussion sections. |
| Study size | 10 | Explain how the study size was arrived at  **Sample size:** The risk of advanced adenoma and advanced neoplasia according to familial- or average-risk was the main study outcome measure. As the familial-risk population comprised individuals with different neoplastic risk, we estimated the sample size needed to yield sufficient statistical power for significant results with respect to the main outcome measure in the least numerous subgroup, i.e. FDR with two relatives diagnosed with colorectal cancer at any age. Considering a ratio of 10:1 for one versus two relatives affected, an advanced adenoma detection rate of 7.8% in individuals with one FDR and 14.7% in those with two FDR with colorectal cancer, with an alpha error of 0.05 (two-sided) and a beta error of 0.10, the number of individuals having two FDR with colorectal cancer required was 235. FDR having one family member with colorectal cancer diagnosed at <60 or ≥60 years were analyzed separately, as an age threshold of 60 years in the index-case is considered a colorectal cancer risk factor for their relatives . |
| Quantitative variables | 11 | Explain how quantitative variables were handled in the analyses. If applicable, describe which groupings were chosen and why.  This information is described in the Statistical Methods. |
| Statistical methods | 12 | (*a*) Describe all statistical methods, including those used to control for confounding |
|  |  | (*b*) Describe any methods used to examine subgroups and interactions |
|  |  | (*c*) Explain how missing data were addressed |
|  |  | (*d*) *Cohort study*—If applicable, explain how loss to follow-up was addressed  *Case-control study*—If applicable, explain how matching of cases and controls was addressed  *Cross-sectional study*—If applicable, describe analytical methods taking account of sampling strategy |
|  |  | (*e*) Describe any sensitivity analyses  Between-group differences in the risk of neoplastic lesions with respect to both overall or colon side-specific colorectal neoplasia detection rates were analyzed by multinomial logistic regression analysis when considering the 4-level categorical variables (normal colonoscopy, non-advanced adenoma, advanced adenoma, and cancer). In these analyses, the most severe colonoscopic finding was represented as an independent category. Binary logistic regression analysis was used when considering another colonoscopic findings included in the previous 4-level categorical variable.  All logistic regression analyses were adjusted for age, gender and center and reported as odds ratios (OR) with 95% confidence intervals (CI) .  A logistic regression model was developed to predict the probability of advanced neoplasia according to age, gender and familial groups. The observed frequencies and probabilities predicted from the regression equation were graphically represented stratifying individuals in 10-year subsets (20 to 70 for the affected familial groups, 50 to 70 for the control group) and by gender.  The analysis of resources, based on the number of colonoscopies needed to detect one advanced neoplasia, was performed by inverse marginal probability estimated by binary logistic regression analysis , adjusted for age and center. All analyses were performed using SPSS version 15.0 (SPSS Inc., Chicago, IL) and STATA version 13.1 (Stata Corp, Texas, USA) software. All statistical tests were two-sided, and P values <0.05 were considered statistically significant. |

Continued on next page

| Results | | |
| --- | --- | --- |
| Participants | 13* | (a) Report numbers of individuals at each stage of study—eg numbers potentially eligible, examined for eligibility, confirmed eligible, included in the study, completing follow-up, and analysed  Overall, 8,498 individuals were assessed for eligibility, of whom 4,175 (49.1%) belonged to the familial-risk cohort and 4,323 (50.9%) to the control cohort. Of these, 1,160 (27.8%) individuals having FDR with colorectal cancer and 1,285 (29.7%) average-risk individuals were excluded (Fig. 1). Finally, 3,015 FDR of patients with colorectal cancer and 3,038 average-risk individuals were included. |
|  |  | (b) Give reasons for non-participation at each stage  This information is detailed in Figure 1. |
|  |  | (c) Consider use of a flow diagram  This information is detailed in Figure 1. |
| Descriptive data | 14* | (a) Give characteristics of study participants (eg demographic, clinical, social) and information on exposures and potential confounders  Demographic data of the familial-risk cohort are shown in Table 1. The number of index-cases was 2,474 (median age 66.2 years). Of these, 1,399 (56.5%) were male and 786 (31.7%) were aged <60 years at diagnosis of colorectal cancer. Siblings with colorectal cancer predominated among individuals having two FDR affected compared to individuals having only one FDR with colorectal cancer (77.7% vs 25.5%, P<0.001) (Table 1). Compared to average-risk individuals, mean age of the familial-risk cohort was lower (51.8 ± 9.0 vs 59.3 ± 5.5 years, P<0.001), as was the proportion of men (41.3% vs 47.6%, P<0.001), as shown in Table 1. |
|  |  | (b) Indicate number of participants with missing data for each variable of interest |
|  |  | (c) *Cohort study*—Summarise follow-up time (eg, average and total amount) |
| Outcome data | 15* | *Cohort study*—Report numbers of outcome events or summary measures over time |
|  |  | *Case-control study—*Report numbers in each exposure category, or summary measures of exposure |
|  |  | *Cross-sectional study—*Report numbers of outcome events or summary measures  This information is detailed in the Main results section. |
| Main results | 16 | (*a*) Give unadjusted estimates and, if applicable, confounder-adjusted estimates and their precision (eg, 95% confidence interval). Make clear which confounders were adjusted for and why they were included  **Risk of Advanced Neoplasia**: Colonoscopy findings are shown in Fig 1. In individuals having two FDR with colorectal cancer, compared to average-risk individuals, we observed a higher prevalence and risk of non-advanced adenoma (OR 1.40; 95% CI 1.02 to 1.92, P=0.03), advanced adenoma (OR 2.13; 95% CI 1.42 to 3.19, P<0.001), ≥3 non-advanced adenomas (OR 2.16, 95% CI 1.21 to 3.84, P=0.01) and advanced neoplasia (OR 1.90; 95% CI 1.36 to 2.66, P<0.001) than in average-risk individuals, after adjusting for age, sex and center (Table 2). In contrast, the prevalence and risk of advanced neoplasia was similar to that of the controls in individuals having only one FDR with colorectal cancer diagnosed at ≥60 years (OR 1.03; 95% CI 0.83 to 1.27, P=0.77) or <60 years (OR 1.19; 95% CI 0.90 to 1.58, P=0.20), as shown in Table 2.  Since average-risk individuals were significantly older than individuals with FDR with colorectal cancer, we next stratified the results by age performing an interaction statistical test between dichotomized age (50-59 vs 60-69 years-old) and individual’s group for colonoscopy findings (see S1 Table). Overall, this test was not statistically significant (p=0.154) in the multinomial logistic regression analyses, indicating that despite the age difference between the two cohorts, age was not a main effect modification factor in the study. |
|  |  | (*b*) Report category boundaries when continuous variables were categorized |
|  |  | (*c*) If relevant, consider translating estimates of relative risk into absolute risk for a meaningful time period |
| Other analyses | 17 | Report other analyses done—eg analyses of subgroups and interactions, and sensitivity analyses  The probability of advanced neoplasia increased with age in both cohorts, as expected (Fig. 2). The prevalence and risk of advanced neoplasia was markedly greater in men than in women in all groups (Table 3) and at each age interval (Fig. 2).  Men and women having one FDR with colorectal cancer consistently showed a similar probability of advanced neoplasia than average-risk men and women, respectively (Fig. 2). Interestingly, after the age of 50 years, advanced neoplasia was over two-fold higher (OR 2.50; 95% CI 1.36-2.66, P<0.001) and developed at least ten years earlier in men compared to women in the subgroup of individuals having two FDR with colorectal cancer, (Fig. 2 and Table 4).  Individuals having two FDR with colorectal cancer showed a significantly higher prevalence and risk of advanced adenomas than average-risk individuals both in distal (OR 2.08; 95% CI 1.35 to 3.19, P=0.001) and proximal colon (OR 1.92; 95% CI 1.08 to 3.40, P=0.026) (Table 5). No differences in the prevalence and risk of advanced adenomas categorized by location were observed for individuals having only one FDR with colorectal cancer compared to average-risk individuals.  **Analysis of resources:** The number of colonoscopies needed to detect one advanced neoplasia was 7 (95% CI 5.4 to 10) in individuals having two FDR with colorectal cancer, 10.6 (95% CI 9.2 to 12.6) in individuals having only one FDR with colorectal cancer diagnosed at <60 years, 9 (95% CI 7.2 to 11.6) or at ≥60 years, and 11.6 (95% CI 10.4 to 13) in average-risk individuals. Approximately two-fold fewer colonoscopies were needed to detect one advanced neoplasia in men than in women in all groups (Table 6). |
| Discussion | | |
| Key results | 18 | Summarise key results with reference to study objectives  The current study adds new insight into the risk and probability of colorectal neoplasia in the familial-risk population. Men and women having two FDR with colorectal cancer showed a significantly greater risk of advanced adenomas and advanced neoplasia than their respective controls. In contrast, men and women having one FDR with colorectal cancer showed a similar risk of these lesions to average-risk individuals, regardless of index-case age at diagnosis of colorectal cancer. The risk of advanced neoplasia was almost three-fold higher and appeared at least ten years earlier in men than in women in both cohorts. |
| Limitations | 19 | Discuss limitations of the study, taking into account sources of potential bias or imprecision. Discuss both direction and magnitude of any potential bias  The study also has certain limitations. First, although average-risk individuals were consecutively included in the COLONPREV study this was not the case in the familial-risk cohort. Fifty five percent of the FDR were consecutively included in two prospective studies, whereas the remaining FDR were not. However, demographic characteristics and risk of advanced neoplasia were similar in FDR regardless of whether they were included consecutively or not (data not shown). This potential bias was also minimized by performing a logistic regression analysis, controlling for confounding factors. Second, due to the study design, there was a substantial difference in age between individuals with family history of colorectal cancer and average risk individuals. However, both logistic regression analyses and stratification by age suggested that age was not a main confounding factor in this study (S1 Table). Third, other confounding factors such as nonsteroidal anti-inflammatory drugs, acetylsalicylic acid, smoking, obesity, and diet were not recorded and they could conceivably have influenced the results. Fourth, unfortunately, the prevalence of serrated polyps was not recorded, because at the time the present study was initiated, classification of serrated polyps was still under debate. In fact, only recently clinical guidelines recommend colonoscopy surveillance for individuals with serrated polyps . Fifth, colorectal cancer family history was obtained by interview and therefore could be underreported . Additionally, the effect of the number of colorectal cancers in relation to the family size could not be analyzed. Sixth, although adenoma detection rate is widely used as colonoscopy quality indicator, unfortunately this information was not available. Since the centers that provided individuals in the average-risk group (COLONPREV) and the ones that recruited individuals in the familial-risk cohort are virtually the same, we do not expect differences in colonoscopy quality between the two cohorts. Finally, some of the individuals included may have been members of families with Lynch syndrome, since we did not systematically exclude DNA mismatch repair deficiency in all cases with colorectal cancer. However, the overall results should not be greatly affected since this possibility would only involve a small number of individuals. |
| Interpretation | 20 | Give a cautious overall interpretation of results considering objectives, limitations, multiplicity of analyses, results from similar studies, and other relevant evidence  Advanced adenoma and early colorectal cancer are surrogate endpoints of colorectal cancer screening as the detection and treatment of these lesions is associated with a significant reduction of colorectal cancer incidence and mortality . Based on this principle, and on population-based studies reporting that FDR of patients with colorectal cancer have a higher relative risk of developing the disease than the general population , current guidelines recommend screening colonoscopy every five or ten years, depending on whether colorectal cancer in the index-case is diagnosed at <60 or ≥60 years. However, there is no clear evidence that the natural history of pre-cancerous lesions and cancer differs between familial- and average-risk populations. In this regard, the risk of adenoma recurrence is more related to the characteristics of the neoplasia at baseline colonoscopy and to demographic data (age and gender) than to family history, suggesting that screening intervals in individuals with familial colorectal cancer could be extended beyond 5 years, as most guidelines recommend .  Previous case-control studies have shown contradictory results with respect to adenoma prevalence in familial colorectal cancer. At least two studies have reported a similar prevalence of adenomas in individuals having one FDR with colorectal cancer compared with average-risk individuals. In contrast, other studies have shown an increased prevalence of advanced adenomas in FDR of patients with colorectal cancer compared with average-risk individuals . However, some of these studies had important methodological flaws: first, the control group was small in most of them and frequently inappropriate, including autopsies , symptomatic patients , or volunteers paying for screening colonoscopy . Second, the small sample size did not allow stratification according to the number of FDR affected . Third, their retrospective design did not allow for ascertaining the indication for colonoscopy and exclusion of patients with hereditary colorectal cancer syndromes. Finally, high quality colonoscopy was not specifically assessed in many studies .  Our study apparently solved these drawbacks, corroborating that among relatives of patients with colorectal cancer, only those with two FDR affected showed a marked increase in the prevalence and risk of advanced neoplasia compared with average-risk individuals in both the proximal and the distal colon. However, the risk of advanced neoplasia in individuals having only one FDR with colorectal cancer diagnosed before or after the age of 60 years was similar to that of average-risk individuals. These findings are in line with the results of a nested study performed within the randomized controlled Prostate, Lung, Colorectal, and Ovarian (PLCO) cancer screening trial of flexible sigmoidoscopy versus usual care, showing that men and women having two FDR with colorectal cancer had a two fold increased risk of incident colorectal cancer, whereas those with one FDR affected were not associated with an increased risk in colorectal cancer incidence or mortality, regardless of their age at the time of diagnosis .  Interestingly, we observed that individuals with 2 FDR had also an increased risk of both non-advanced and advanced-adenomas. In addition, men showed an almost three-fold higher risk of advanced neoplasia than women in all groups, and advanced neoplasia appeared at least 10 years earlier in men than in women. It is interesting to note that the number of colonoscopies needed to detect one advanced neoplasia was two-fold higher in women than in men at all ages in both cohorts. Taken together, our results support the notion that screening colonoscopy may be delayed at least ten years in women having one or even two FDR with colorectal cancer, as has been shown for women in the average-risk population .  Our study has several strengths. First, participants were recruited with strict selection criteria regarding colonoscopy quality and inclusion age. Only individuals with a first lifetime complete colonoscopy were eligible and an upper age limit of 69 years was established to minimize the effect of age on the risk of advanced neoplasia. Second, colonoscopies were performed by the same endoscopists who applied the same quality criteria in both cohorts. Third, to our knowledge, this is the largest study to compare the risk of advanced neoplasia between asymptomatic FDR of patients with colorectal cancer and average-risk individuals, thus allowing an accurate estimation of this parameter according to the number of close relatives with colorectal cancer, gender and index-case age at diagnosis of the disease. |
| Generalisability | 21 | Discuss the generalisability (external validity) of the study results  Our findings suggest that screening guidelines for the management of familial colorectal cancer, if not adjusted for the number of relatives affected and sex, may substantially overestimate the prevalence of advanced neoplasia, particularly in men and women having one FDR with colorectal cancer and in women having two FDR with colorectal cancer diagnosed before the age of 50. In fact, the same screening strategy as that for average-risk individuals could be recommended to men and women having only one FDR with colorectal cancer, but starting at the age of 40 or 45 years, in line with the results of previous studies , thus avoiding overuse of screening colonoscopy. In support of this recommendation, there are two recent prospective studies demonstrating that fecal immunochemical testing is as effective as colonoscopy to detect advanced neoplasia in familial colorectal cancer . In contrast, the higher prevalence and earlier presentation of advanced neoplasia in men having two FDR with colorectal cancer suggest that men have higher genetic penetrance, thus supporting screening colonoscopy as from the age of 40, whereas it could be delayed until the age of 50 or more in women with two FDR.  In conclusion, our study demonstrates that the risk and predicted probability of advanced neoplasia are markedly increased in individuals having two FDR with colorectal cancer compared to average-risk individuals, but not in individuals having only one FDR with colorectal cancer, regardless of when they were diagnosed. Our results indicate that men having two FDR with colorectal cancer should undergo an early screening colonoscopy, whereas individuals having only one FDR with colorectal cancer could be recommended to undergo the same screening strategy as the average-risk population, but starting at an earlier age. |
| Other information | | |
| Funding | 22 | Give the source of funding and the role of the funders for the present study and, if applicable, for the original study on which the present article is based  This study was supported by grants from “Fundación Canaria para la Investigación Sanitaria” (FUNCIS) (P21-02) and “Caja de Canarias”, and from “Asociación Española contra el Cáncer (Fundación Científica and Junta de Barcelona)”, the “Instituto de Salud Carlos III (PI08-90717; PI10-00384; PI13-00719)”, FEDER funds, and the “Agència de Gestió d’Ajuts Universitaris i de Recerca (GRC 2014SGR135)”. CIBERehd is funded by the Instituto de Salud Carlos III. In the Basque Country, the study received additional support with grants from Obra Social de Kutxa, Diputación Foral de Gipuzkoa (DFG 07-5), Departamento de Sanidad del Gobierno Vasco, EITB-Maratoia (BIO 07-CA-19) y Acción Transversal contra el Cáncer del CIBERehd (2008). In Galicia, this work was supported by Dirección Xeral de Innovación e Xestión da Saúde Pública, Conselleria de Sanidad de la Xunta de Galicia. OC-Micro instruments and fecal immunochemical tests were kindly provided by Eiken Chemical Co., Ltd., Japan, and its Spanish representatives, Palex Medical and Biogen Diagnóstica; none of them were involved in the design of the study or in the analysis or interpretation of results. |

*Give information separately for cases and controls in case-control studies and, if applicable, for exposed and unexposed groups in cohort and cross-sectional studies.

**Note:** An Explanation and Elaboration article discusses each checklist item and gives methodological background and published examples of transparent reporting. The STROBE checklist is best used in conjunction with this article (freely available on the Web sites of PLoS Medicine at http://www.plosmedicine.org/, Annals of Internal Medicine at http://www.annals.org/, and Epidemiology at http://www.epidem.com/). Information on the STROBE Initiative is available at www.strobe-statement.org.
